# Synthesis and evaluation of UiO-66@MIP towards norfloxacin in water†

**DOI:** 10.1039/d2ra02726g

**Published:** 2022-07-21

**Authors:** Zixuan Wu, Wanqiong Liu, Sixue Zhang, Zhihua Peng, Yanshan Dong, Zeyu Huang, Mingmin Zhong, Youai Ye, Xiaoping Su, Yong Liang

**Affiliations:** Department of Analytical Chemistry, Faculty of Chemistry, South China Normal University Guangzhou China liangy@scnu.edu.cn; Foshan Sanshui Foshui Water Supply Co., Ltd Foshan China

## Abstract

Norfloxacin (NOX), a kind of quinolone antibiotic, is widely used in disease treatment and the control of human and livestock products. Due to overuse, norfloxacin has become a common organic pollutant in water. We combine the high specific surface area and high stability of metal–organic frameworks with the high selectivity of molecularly imprinted polymers. By grafting a carbon–carbon double bond on the surface of UiO-66–NH_2_, a molecularly imprinted layer is formed on the surface of UiO-66–NH_2_ upon free radical polymerization. The saturated adsorption capacity of UiO-66@MIP reaches 58.01 mg g^−1^. UiO-66@MIP exhibits high adsorption performance in real water samples and its recoveries range from 96.7% to 98.3%, which demonstrates a higher adsorption capacity and recovery than other molecularly imprinted materials and has potential applications in the removal of norfloxacin in real life.

## Abstract 2

In this study, UiO-66@MIP was synthesized to remove NOX. The ability of UiO-66@MIP to adsorb norfloxacin in actual wastewater was also explored.

## Graphical abstract

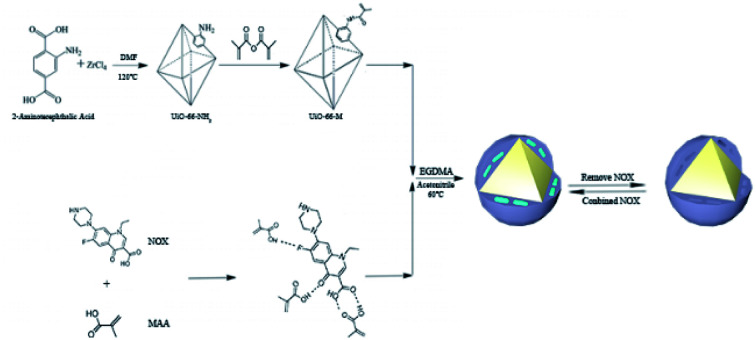

## Introduction

Norfloxacin (NOX), as a broad-spectrum inexpensive antibiotic, is widely used for disease treatment and the control of human and livestock products. The percentage of antibiotics used in China exceeds 50% of the total global use. After being partially metabolized, large amounts of antibiotics are excreted into the environment with stool and urine.^1^ Therefore, antibiotics have been frequently detected in lakes and rivers. Drinking water containing antibiotics for a long time may reduce human immunity, cause an imbalance of intestinal flora and even lead to cancer and teratogenicity. Hence, it is necessary to develop a low-cost, simple and efficient separation and detection method for norfloxacin. At present, solid-phase extraction (SPE) and QuEChERS have been widely used to extract norfloxacin from water; however, they both have high cost and some of them have low adsorption capacity.^2–4^

Molecularly imprinted polymers (MIPs) are polymers that can selectively bind to template targets *via* a key and lock mechanism.^5,6^ Due to its low cost, high stability and high selectivity, molecular imprinting is a common method to increase the selectivity of materials.^7–9^ For instance, the combination of molecular imprinting and quantum dots can be applied to the detection of pollutants and cancer imaging,^10–14^ MIPs coupled with silica are used as filler for solid-phase extraction, and the modification of molecular imprinting on magnetic nanoparticles can greatly improve the efficiency of sample pretreatment.^15–18^

In recent years, metal–organic frameworks (MOFs) have become the focus of many studies owing to their large accessible surface areas, uniformity, tunable pore sizes, chemical modularity, fluorescence and catalytic activity. MOFs and their composite materials are widely used in the fields of separation and enrichment, analysis and detection.^19,20^ However, the selectivity of metal–organic frameworks mainly depends on their specific pores. When facing different targets, it is necessary to design pores similar to the molecular structure of the targets to achieve specific adsorption and separation, which is not flexible in application. Thus, molecular imprinting technology has the potential to improve the selective adsorption capacity of MOFs.^21^ Molecular imprinting has been successfully combined with MOFs by adding template molecules before forming the metal–organic frameworks so that the synthesized metal–organic frameworks have the ability to distinguish template analogs.^22^ Furthermore, metal–organic frameworks with nano-enzyme activity coupled with molecular imprinting have been used as selective colorimetric probes for inorganic compound detection.^23,24^ These molecular imprinting techniques, which can enhance the ability of metal–organic frameworks, can also be applied in practical scenarios.

**Fig. 1:**
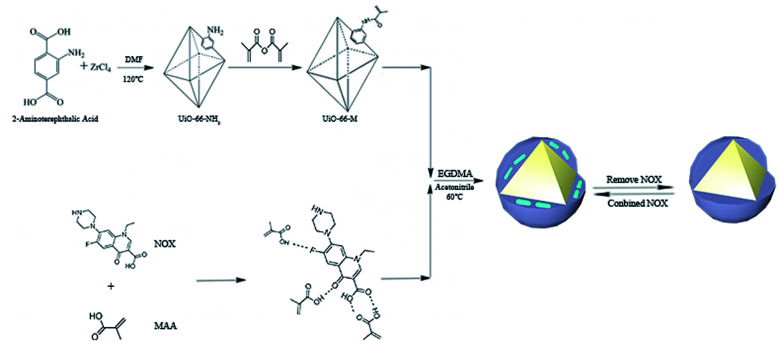
Synthetic process of UiO-66@MIP.

In this study, UiO-66–NH_2_ with high stability was chosen as the carrier of molecular imprinting. After grafting double bonds on the surface, NOX was imprinted on UiO-66–NH_2_ by free-radical polymerization. The synthesized UiO-66@MIP was shown to have high stability and selectivity and a higher adsorption capacity than ordinary molecularly imprinted materials. Convincingly, through real water sample application, UiO-66@MIP exhibits high adsorption performance, with recoveries ranging from 96.7% to 98.3% (Fig. 1).

## Experimental section

### Materials

*N*,*N*-Dimethylformamide (DMF), acetonitrile, methacrylate (MAA), zirconium oxychloride (ZrOCl_2_), azodiisobutyronitrile (AIBN), 2-aminoterephthalic acid (H_2_BDC-NH_2_), ethylene glycol dimethacrylate (EGDMA), norfloxacin (NOX), ciprofloxacin (CIP), tetracycline (TC), sulfadiazine (SD) and acetic acid (HAc) were purchased from Macklin (Shanghai, China). Methanol and ethanol were purchased from Damao Chemical Reagent Factory (Tianjin, China).

### Instrumentation

X-ray diffraction (XRD) measurements were carried out on a Bruker D8 Advance Scattering system (Bruker, Germany). Fourier transform infrared spectra (FT-IR) (4000–400 cm^−1^) in KBr were recorded on a Varian DRX-400 Fourier Transform Spectrophotometer (Agilent Technologies, USA). Thermogravimetric analysis (TGA) was performed using a TG 209 F3 thermal analyzer (Netzsch, Germany) in a N_2_ atmosphere at a heating rate of 5 °C min^−1^. The morphology of UiO-66@MIP was characterized by transmission electron microscopy (TEM) on a JEM-2010HR at 120 kV (JEOL, Japan). The pore size of the materials and their N_2_ adsorption–desorption measurements were analysed with an ASAP-2460 surface area and pore size analyzer (Micromeritics Instrument Corp, USA). HPLC was performed using an LC-20AT LC system coupled with an SPD-20A UV-Vis detector (Shimadzu, Japan).

### Preparation of UiO-66–NH_2_

UiO-66–NH_2_ was prepared according to a published procedure.^23^ Briefly, ZrOCl_2_ (0.78 g) and acetic acid (5.55 mL) were dissolved in DMF (80 mL) with ultrasound for 5 minutes. Then, 2-aminoterephthalic acid was dissolved in this solution. After an additional 5 minutes of ultrasound, deionized water (0.24 mL) was added to the solution. The mixed solution was transferred to a Teflon reactor and heated to 120 °C for 24 h and then cooled to room temperature. The product was repeatedly washed with DMF and ethanol and then dried at 60 °C under vacuum.

### Synthesis of UiO-66–M

The as-synthesized UiO-66–NH_2_ (1 g) was dispersed in dichloromethane (15 mL). After being sonicated for 20 minutes, methacrylic anhydride (2.6 mL) was added to the solution. The whole reaction lasted for 96 h at 25 °C. After the reaction, the precipitate was collected with a centrifuge at 9000 rpm, and washed with dichloromethane 3 times. The product was dried at 45 °C under vacuum.^24^

### Synthesis of UiO-66@MIP

80 mg of UiO-66–M and 50 mL acetonitrile were added to a 100 mL flask. After being sonicated for 10 min, 51 mg NOX and 68 μL MAA were added to the flask. The mixture was stirred for 2 h at room temperature. After the reaction system was heated to 60 °C, 400 μL EGDMA and 70 mg AIBN were added to the solution. The mixture reacted at 60 °C for 24 hours. After the reaction, the precipitate was collected with a centrifuge at 9000 rpm, then washed with methanol/acetic acid (90 : 10, v/v) until the template was removed. Finally, the product was dried at 60 °C under vacuum.

For comparison, the synthetic process of UiO-66@NIP was the same as that of UiO-66@MIP without adding NOX.

### Adsorption capacity of UiO-66@MIP

The adsorption capacity of UiO-66@MIP was studied through static adsorption experiments, dynamic adsorption experiments and selective adsorption experiments.

For the dynamic adsorption experiments, briefly, NOX was prepared in standard solutions with concentrations from 10 mg L^−1^ to 500 mg L^−1^. NOX standard solution was added to a centrifuge tube, and about 9 mg of UiO-66@MIP and UiO-66@NIP were then added separately. The mixture was incubated on a shaker at 500 rpm for 24 hours. Supernatant solutions were collected by centrifugation. The supernatant solutions were detected by UV spectrophotometry at 277 nm and the concentrations of NOX were calculated according to the standard curve for NOX.

The adsorption capacity (*Q*, mg g^−1^) for UiO-66@MIP and UiO-66@NIP were calculated by the following equation:

1 *Q* = (*C*_0_ − *C*) × *V*/*m*

where *C*_0_ (mg L^−1^) is the initial concentration of the NOX standard solutions, *C* (mg L^−1^) is the concentration of the solution after the adsorption is completed, *V* (mL) is the volume of the NOX standard solutions added, and *m* is the mass of UiO-66@MIP or UiO-66@NIP added.

The imprinting factor (*α*) and selectivity factor (*β*) are important standards to measure the performance of molecularly imprinted polymers and non-molecularly imprinted polymers, which can be calculated by the following equation:

2 *α* = *Q*_MIP_/*Q*_NIP_

3 *β* = *α*_1_/*α*_2_

where *Q*_MIP_ and *Q*_NIP_ are the adsorption capacities of UiO-66@MIP and UiO-66@NIP, respectively, *α*_1_ is the imprinting factor of NOX, and *α*_2_ is the imprinting factor of the other test objectives.

For the dynamic adsorption experiments, UiO-66@MIP was weighed in a centrifuge tube. Then, 3 mL of 200 mg L^−1^ solution was added to it. The mixtures were incubated in a shaker separately at 500 rpm from 1 min to 50 min. Supernatant solutions were collected by centrifugation and detected by UV spectrophotometry at 277 nm.

For the selective adsorption experiments, 2 mL NOX, CIP, SD and TC solutions with a concentration of 200 mg L^−1^ were mixed with UiO-66@MIP and UiO-66@NIP. They were incubated on a shaker at 500 rpm for 60 min. After filtering out the precipitate, the supernatant was collected for testing.

### Applications in real samples

The water sample was provided by the Maozi Feng water plant. After adding three different concentrations of norfloxacin standard solution to a 3 mL water sample, UiO-66@MIP was added and the mixture was put into a shaker for 30 minutes. After adsorption, the supernatant was collected using a centrifuge and filtered with a 0.22 μm nylon filter. The content of norfloxacin in the supernatant was detected by high-performance liquid chromatography (HPLC).

The HPLC measurement was performed with a C_18_ column (200 mm × 4.6 mm) using 0.025 mol L^−1^ phosphoric acid/acetonitrile (v/v = 8/2) as the mobile phase with a flow rate of 1 mL min^−1^. The detection wavelength was set to 278 nm and the column temperature was 30 °C.

## Results and discussion

### Structural analyses

**Fig. 2:**
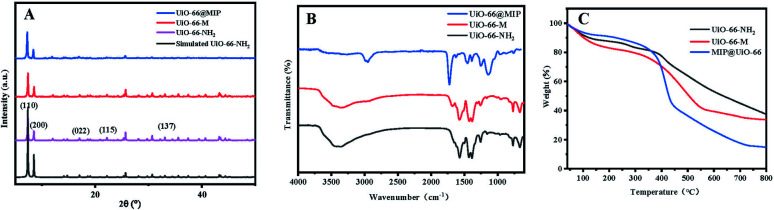
(A) XRD spectra of the studied materials (the black line represents simulated UiO-66–NH_2_; the pink line, UiO-66–NH_2_; the red line, UiO-66–M; and the blue line UiO-66@MIP). (B) FT-IR spectra of the studied materials (black represents UiO-66–NH_2_; red, UiO-66–M; and blue, UiO-66@MIP). (C) Thermogravimetric characterization of UiO-66–NH_2_, UiO-66–M and UiO-66@MIP (black, red and blue, respectively).

In order to find whether the material remains stable before and after modification and polymerization, UiO-66–NH_2_, UiO-66–M and UiO-66@MIP were analysed by powder X-ray diffraction (PXRD). The PXRD patterns are shown in Fig. 2A. As shown, the peaks of the black line at 2*θ* = 7.36°, 8.48°, 17.08°, 22.25° and 33.12° correspond to the (110), (200), (022), (115) and (137) characteristic diffraction peaks of UiO-66, respectively.^25,26^ After the reaction between UiO-66–NH_2_ and methacrylic anhydride, the diffraction peaks of UiO-66–M remained consistent with the original UiO-66, indicating that the crystal structure is not destroyed by the reaction. Due to the modification of molecular imprinting, the PXRD patterns of UiO-66@MIP have a high baseline, and therefore background subtraction and baseline correction were performed on the obtained data. The peak of the obtained pattern is the same as that of the original UiO-66–NH_2_, indicating that even after polymerization, UiO-66–M maintains its original crystal structure.

FT-IR spectroscopy (Fig. 2B) was used to study the composition of UiO-66@MIP and to see whether UiO-66–M and UiO-66@MIP were successfully synthesized. The absorptions at 3461 and 3351 cm^−1^ correspond to the symmetric and asymmetric N–H vibration.^27^ The N–H bending vibration and C–N stretching can be found from 1572 to 1385 cm^−1^.^28^ Compared with the FT-IR spectrum of UiO-66–NH_2_, UiO-66–M has a new absorption peak at 1673 cm^−1^, which is attributed to the characteristic absorption peak of C=C. The spectrum shows that UiO-66–M is successfully synthesized. Due to the formation of the molecularly imprinted layer, the FT-IR spectrum of UiO-66@MIP is mainly composed of the 2950 cm^−1^ C–H absorption peak and 1716 cm^−1^ C=O absorption peak.^29^ In other words, UiO-66–M and UiO-66@MIP have been successfully synthesized.

Fig. 2C shows the thermogravimetric characterization of UiO-66–NH_2_, UiO-66–M and UiO-66@MIP. The thermogravimetric curves of UiO-66–NH_2_ and UiO-66–M are roughly similar. The mass reduction before 100 °C is attributed to the loss of solvent and water. When the temperature rises to 270 °C, the material begins to decompose, generating CO, CO_2_ and zirconia. The weight-loss rate of UiO-66–NH_2_ is 62.4%. In terms of combustion residue, UiO-66–NH_2_ is slightly higher than UiO-66–M, which also proves that the double bond has been successfully modified. The blue line (Fig. 2C) represents UiO-66@MIP; its thermogravimetric curve is obviously different from that of UiO-66–NH_2_ and its weight-loss rate is 85.2%, which proves that norfloxacin has been successfully imprinted on the surface of UiO-66–NH_2_.

**Fig. 3:**
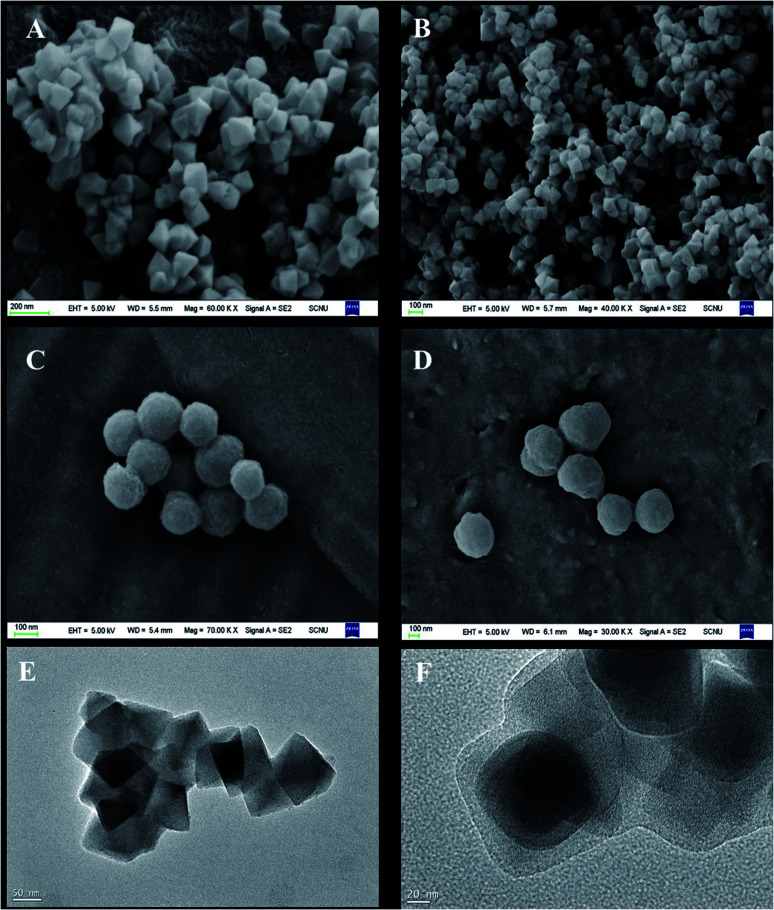
SEM images of (A) UiO-66–NH_2_, (B) UiO-66–M, (C) UiO-66@MIP, and (D) UiO-66@NIP. TEM images of (E) UiO-66–NH_2_ and (F) UiO-66@MIP.

Fig. 3 presents the SEM and TEM images of the studied materials. UiO-66–NH_2_ presents a typical octahedral structure. After reacting with methacrylic anhydride, the crystal structure of UiO-66–M does not change. This result echoes the previous XRD result. Due to the modification of the molecularly imprinted layer, the structure of UiO-66@MIP is transformed from the original octahedra to a sphere. It can be seen from the TEM image (Fig. 3F) that UiO-66–NH_2_ is encapsulated by the imprinted layer. The thickness of the imprinted layer is about 20 nm.

### Optimization of adsorption conditions

#### Optimization of the ratio of template functional monomers and crosslinking agents

**Fig. 4:**
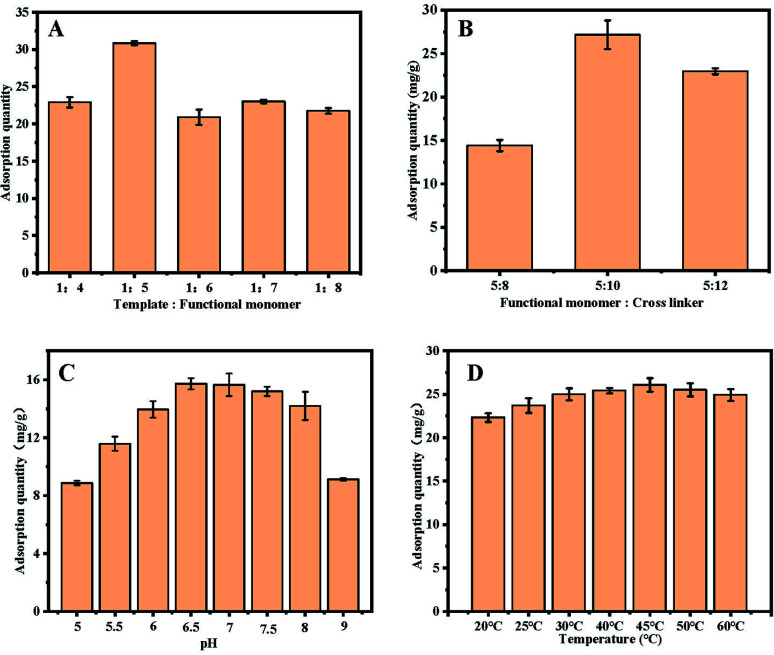
(A) The result of the optimization of the ratio of the template and functional monomers. (B) The result of the optimization of the ratio of functional monomers and crosslinking agents. (C) The effect of pH on the adsorption of UiO-66@MIP. (D) The effect of temperature on the adsorption of UiO-66@MIP.

The ratio of functional monomer to crosslinking agent plays an important role in the adsorption capacity of molecularly imprinted materials. In order to obtain UiO-66@MIP with a large adsorption capacity, the ratio of functional monomer to template and the ratio of functional monomer to crosslinking agent were optimized. When studying the ratio of functional monomer to template, the ratio of the functional monomer to crosslinking agents is 5 : 10. Because some amount of functional monomer cannot be fully pre-polymerized with the template, the adsorption capacity will be low. With more functional monomers, the non-specific adsorption will increase, which also affects the adsorption capacity of UiO-66@MIP. As seen from Fig. 4A, when the ratio of functional monomer to template is 5 : 1, UiO-66@MIP has better adsorption performance. While exploring the ratio of functional monomer to crosslinking agents, the ratio of functional monomer to template is 5 : 1. As a result (Fig. 4B), if the ratio is too low, the imprinted polymer cannot be formed, and if the ratio is too high, the polymerization process will become violent and have side effects on the pores of the imprinted polymer. When the ratio of the functional monomer to crosslinking agents is 5 : 10, UiO-66@MIP exhibits the best adsorption capacity.

#### Optimization of adsorption

Since external conditions have a certain influence on the adsorption capacity of UiO-66@MIP, our study focuses on investigating the influence of pH and temperature on the adsorption of UiO-66@MIP. In order to make the pH of the adsorption conditions consistent with that of environmental water samples, we chose to explore the pH range of 5–9. Norfloxacin is a typical amphoteric compound, so pH has a great influence on the state of norfloxacin. According to previous research, the p*K*_a1_ and p*K*_a2_ of norfloxacin are 6.20 and 8.70, respectively; when the pH is greater than 8.70, norfloxacin will mainly exist in the anionic form, and when the pH is lower than 6.20, norfloxacin will be in its cationic form.^30–33^ Thus, when the pH of the solution is greater than 8.7 but lower than 6.2, norfloxacin and the recognition site of the molecular imprint will experience electrostatic repulsion, thereby hindering its interaction with UiO-66@MIP. On the contrary, when the pH is between 6.2 and 8.7, norfloxacin is in a neutral state, and more easily interacts with UiO-66@MIP through hydrogen bonding. This condition also meets the application requirements in real life. In order to study the accuracy of the adsorption performance, pH 7 was selected as the subsequent experimental condition.

Fig. 4C proves that temperature does not have a great influence on the adsorption of UiO-66@MIP. For the convenience of follow-up research, 35 °C is chosen as the adsorption temperature.

#### Static adsorption experiments

**Fig. 5:**
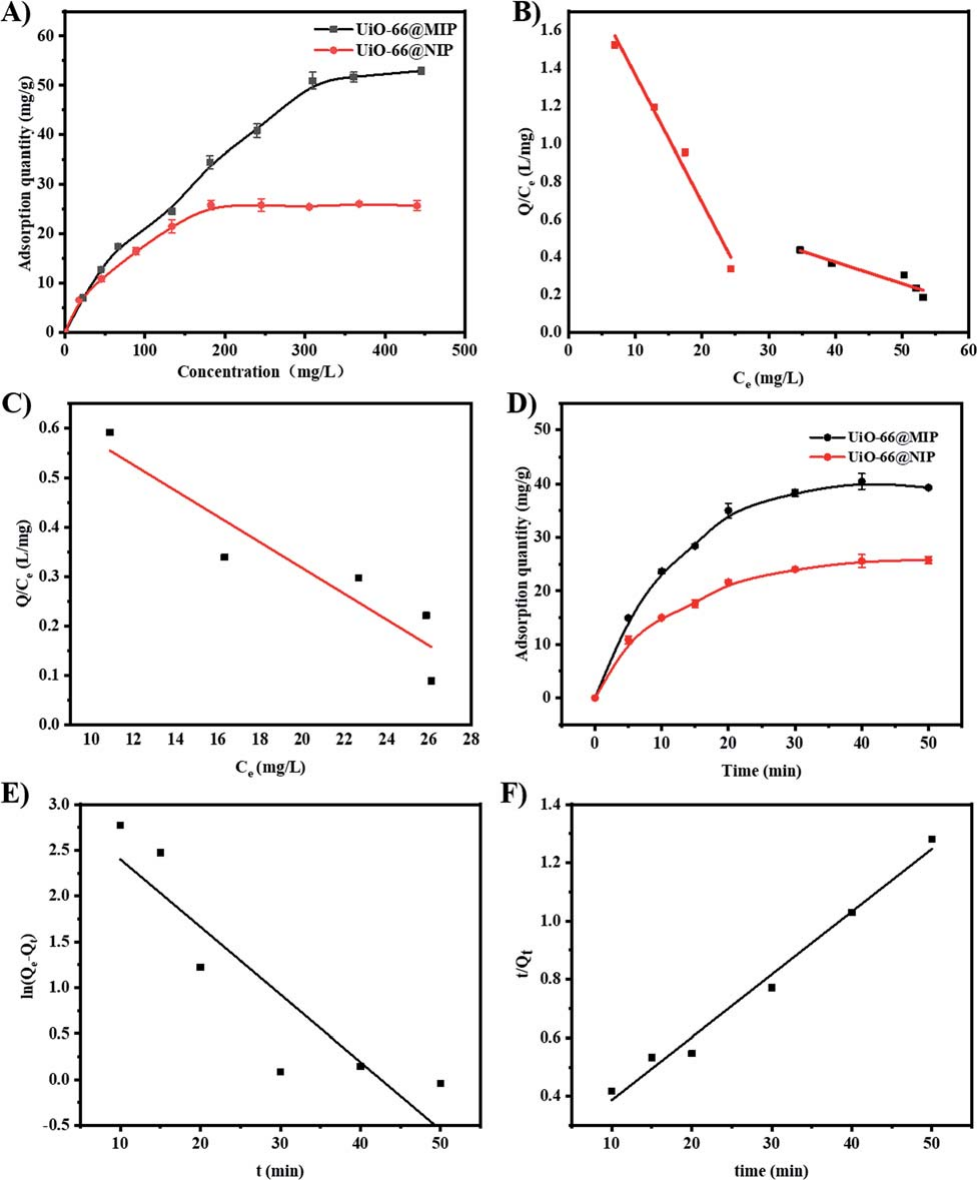
(A) The static adsorption curves of UiO-66@MIP and UiO-66@NIP. (B) Scatchard diagram of UiO-66@MIP. (C) Scatchard diagram of UiO-66@NIP. (D) Dynamic adsorption curves of UiO-66@MIP and UiO-66@NIP. (E) Pseudo-first-order kinetic models. (F) Pseudo-second-order kinetic models.

The static adsorption curves of UiO-66@MIP and UiO-66@NIP were studied at concentrations of norfloxacin in the range of 0–450 mg L^−1^. As shown in Fig. 5A, due to the absence of imprinting sites, the UiO-66@NIP adsorption capacity reaches saturation at a concentration of 200 mg L^−1^. In contrast, when the concentration of norfloxacin was more than 300 mg L^−1^, the adsorption capacity of UiO-66@MIP gradually approaches equilibrium, which shows that the imprinting sites on UiO-66@MIP have reached saturation. The static adsorption curves of UiO-66@MIP and MIP are shown in Fig. S1;† since UiO-66–NH_2_ acts as the carrier of molecular imprinting, the adsorption capacity of UiO-66@MIP is much higher than that of MIP without UiO-66–NH_2_.

As shown in Table S1,† the adsorption process of UiO-66@MIP and UiO-66@NIP for NOX fits well with the Langmuir and Freundlich isotherm models. The model equations are expressed in (4) and (5), respectively.

4 *Q*_e_ = *K*_L_*Q*_m_*C*_e_/(1 + *K*_L_*C*_e_)

5 log *Q*_e_ = (1/*n*)log *C*_e_ + log *K*_F_

where *C*_e_ (mg L^−1^) represents the equilibrium concentration of NOX, *Q*_e_ (mg g^−1^) and *Q*_m_ (mg g^−1^) are the equilibrium adsorption and maximum adsorption capacity of the adsorbents, respectively, *K*_L_ and *K*_F_ are the Langmuir and Freundlich constants, respectively, and 1/*n* represents the heterogeneity index.

The results show that the Freundlich isotherm model is better than the Langmuir. Additionally, the log *K*_F_ value of UiO-66@MIP was higher than that of UiO-66@NIP, indicating that UiO-66@MIP has better adsorption capability than UiO-66@NIP. Moreover, the Freundlich constants (1/*n*) are between 0.386 and 0.631, signifying that the binding sites are distributed on the surface of the adsorbents. Above all, the adsorption of NOX by UiO-66@MIP can be attributed to multilayer adsorption over the heterogeneous surface with non-uniform adsorption sites.^34^

The Scatchard equation is an important criterion for evaluating the static adsorption of UiO-66@MIP and UiO-66@NIP.

6 *Q*/*C*_e_ = (*Q*_m_ − *Q*)/*K*_d_

where *Q*_m_ represents the maximum adsorption capacity of the material, *K*_d_ is the dissociation constant and *C*_e_ is the equilibrium concentration of norfloxacin in the solution when the adsorption reaches equilibrium.^36^

Fig. 5B shows that the Scatchard diagram of UiO-66@MIP is composed of two different linear equations, which illustrates that MIP has two different binding sites. One part of the linear regression equation is *y* = 2.038 − 0.0674*x*, when the initial concentration is in the range of 20 mg L^−1^ to 200 mg L^−1^. When the concentration is from 200 mg L^−1^ to 450 mg L^−1^, the other part of the linear regression equation is *y* = 0.731 − 0.0126*x*, which corresponds to a *Q*_m_ value of 58.01 mg g^−1^. Compared to UiO-66@MIP, the Scatchard diagram of UiO-66@NIP (Fig. 5C) has only one linear segment whose equation is *y* = 0.738 − 0.0301*x*, which corresponds to a *Q*_m_ value of 24.52 mg g^−1^.

#### Dynamic adsorption experiments

The dynamic adsorption curves of UiO-66@MIP and UiO-66@NIP are shown in Fig. 5D. When the initial concentration of norfloxacin is 200 mg L^−1^, UiO-66@MIP reaches adsorption equilibrium at 30 min. Compared with UiO-66@MIP, UiO-66@NIP reaches adsorption equilibrium at 30 min; its adsorption capacity is lower than that of UiO-66@MIP, which is attributed to the absence of specific binding sites.

In this study, pseudo-first-order and pseudo-second-order kinetic models are used to investigate the dynamic adsorption process of UiO-66@MIP. The relevant equations are as follows:

7 ln(*Q*_e_ − *Q*_*t*_) = ln *Q*_e_ − *k*_1_*t*

8 *t*/*Q*_*t*_ = 1/*k*_2_*Q*_e_^2^ + *t*/*Q*_*t*_

where *Q*_e_ and *Q*_*t*_ are the amount of NOX (mg g^−1^) absorbed at the equilibrium time *t* (min) and any time *t* (min), respectively. *k*_1_ and *k*_2_ are the rate constants for the pseudo-first-order and pseudo-second-order kinetic models, respectively.

As shown in Fig. 5E and F, the pseudo-second-order model has a higher determination coefficient (*R*^2^ = 0.98) than the pseudo-first-order model (0.81), indicating that the pseudo-second-order model is suitable to describe the adsorption process of NOX and the adsorption process is more inclined to chemical adsorption.^35^

#### Selectivity of UiO-66@MIP

**Fig. 6:**
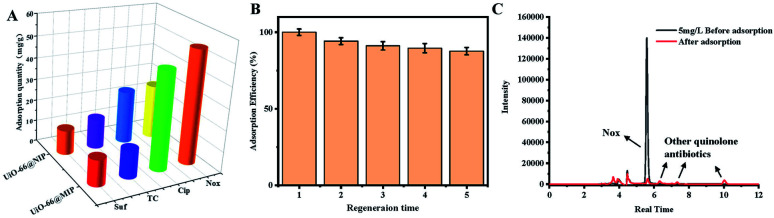
(A) Selectivity of UiO-66@MIP. (B) Reusability of UiO-66@MIP. (C) After adding 5 mg L^−1^ NOX to the water sample, the comparison of HPLC signals before and after adsorption.

Norfloxacin, its structural analog ciprofloxacin (CIP) and other kinds of antibiotics (SD, TC) were chosen to evaluate the selectivity of UiO-66@MIP. As shown in Fig. 6A, UiO-66@MIP shows ultra-high selectivity in the adsorption of other types of antibiotics. In contrast, UiO-66@NIP shows obvious non-specific adsorption to these antibiotics, which is attributed to the absence of molecular imprinting recognition sites. However, due to the small structural difference between ciprofloxacin and norfloxacin, when UiO-66@MIP interacts with ciprofloxacin, it has a priority of occupying the binding sites, which leads to a higher adsorption capacity, but the adsorption capacity is still lower than that of UiO-66@MIP for norfloxacin. This result is consistent with previously reported experimental results.^37,38^ To sum up, the imprinting factors (*α*) of UiO-66@MIP for NOX, CIP, SD, and TC are 2.09, 1.86, 0.94, and 1.07, respectively, while the selectivity factors (*β*) of UiO-66@NIP for CIP, SD, and TC are 1.12, 2.22, and 1.95, respectively. In other words, this selectivity experiment proves that molecularly imprinted sites are generated on the surface of UiO-66–NH_2_.

#### Reusability of UiO-66@MIP

The reusability of UiO-66@MIP is an important indicator of its applicability in the actual detection of norfloxacin. To evaluate the reusability, UiO-66@MIP was mixed with 3 mL norfloxacin solution with a concentration of 90 mg L^−1^ at 450 rpm for 30 min. After adsorption, the supernatant was collected by centrifugation, and the concentration of norfloxacin remaining in the solution was detected by UV spectrophotometry. For UiO-66@MIP, a solution of methanol/acetic acid (90 : 10, v/v) was used to elute NOX. The above process was repeated 5 times. After five cycles, UiO-66@MIP still maintains a high adsorption efficiency, which only decreases by 12%. This result shows that UiO-66@MIP has excellent stability and reusability.

#### Application in real samples

In order to verify the adsorption capacity of UiO-66@MIP in real samples, we found water samples containing norfloxacin. As shown in Fig. 6C, the signal of norfloxacin appears together with the signal of other substances in water, while after adsorption by UiO-66@MIP, the peak of norfloxacin weakens but the signals of other substances are enhanced. We can also find peaks from other types of quinolone antibiotics.

We also carried out spiking and recovery experiments. As shown in Table S2,† the recoveries range from 96.7% and 100.9% with an RSD below 4.4%. Compared with the previously reported materials listed in Table 1, UiO-66@MIP has higher adsorption capacity and better regeneration, which indicates that UiO-66@MIP has the potential to be used in real life to remove norfloxacin.

**Table 1:** Comparison of different materials for the adsorption and determination of NOX

Samples	Detection methods	Relative material	Adsorption capacity (mg g^−1^)	LOD (ng mL^−1^)	Recovery (%)	RSD (%)	Reference
Human urine	HPLC-FLD	Water-compatible MIP	8.6	1.9	53–88	1–10	39
Lake water	HPLC-MS	Dual-template MIP	32	0.22	81.2–97	0.9–5.6	40
Lake water	HPLC-MS	MIP-hollow fibers	4.9	0.9	13.9–18	2.1–4.3	41
Lake water	HPLC-UV	Magnetic MIP	27.04	6	90.9–97.1	1.2–6.8	37
Lake water	HPLC-UV	UiO-66@MIP	58.01	7.5	96.7–98.3	1.9–5.5	This work

## Conclusions

In this study, we used a metal–organic framework as a carrier for molecular imprinting and synthesized UiO-66@MIP that can specifically adsorb norfloxacin. FT-IR, XRD, SEM and other characterization experiments prove the successful synthesis of UiO-66@MIP. Through a series of optimizations, the saturated adsorption capacity of UiO-66@MIP reached 58.01 mg g^−1^, and UiO-66@MIP can also actively separate norfloxacin from mixed solutions. This method can be a new route for the efficient and effective separation of norfloxacin in water and other samples.

## Conflicts of interest

The authors declare that there are no conflicts of interest.

## Supplementary Material

RA-012-D2RA02726G-s001

## References

[cit1] Wang G., Zhao D., Kou F., Ouyang Q., Chen J., Fang Z. (2018). Chem. Eng. J..

[cit2] Cao Y., Huang Z., Luo L., Li J., Li P., Liu X. (2021). Food Chem..

[cit3] Fang L., Miao Y., Wei D., Zhang Y., Zhou Y. (2021). Chemosphere.

[cit4] Maia A. S., Paíga P., Delerue-Matos C., Castro P. M., Tiritan M. E. (2020). Environ. Pollut..

[cit5] BelBruno J. J. (2018). Chem. Rev..

[cit6] Cecchini A., Raffa V., Canfarotta F., Signore G., Piletsky S., MacDonald M. P., Cuschieri A. (2017). Nano Lett..

[cit7] Zhang N., Zhang N., Xu Y., Li Z., Yan C., Mei K., Ding M., Ding S., Guan P., Qian L. (2019). Macromol. Rapid Commun..

[cit8] Turiel E., Martín-Esteban A. (2019). TrAC, Trends Anal. Chem..

[cit9] Niu M., Pham-Huy C., He H. (2016). Microchim. Acta.

[cit10] Chantada-Vázquez M. P., Sánchez-González J., Peña-Vázquez E., Tabernero M. J., Bermejo A. M., Bermejo-Barrera P., Moreda-Piñeiro A. (2016). Anal. Chem..

[cit11] Haupt K., Medina Rangel P. X., Bui B. T. S. (2020). Chem. Rev..

[cit12] Iwanowska A., Yusa S. I., Nowakowska M., Szczubiałka K. (2016). J. Sep. Sci..

[cit13] Yang Q., Li J., Wang X., Xiong H., Chen L. (2019). Anal. Chem..

[cit14] Xie X., Hu Q., Ke R., Zhen X., Bu Y., Wang S. (2019). Chem. Eng. J..

[cit15] Dil E. A., Doustimotlagh A. H., Javadian H., Asfaram A., Ghaedi M. (2021). Talanta.

[cit16] Sadegh N., Asfaram A., Javadian H., Haddadi H., Sharifpour E. (2021). J. Chromatogr. B: Anal. Technol. Biomed. Life Sci..

[cit17] Cai T., Zhou Y., Liu H. (2021). J. Chromatogr. A.

[cit18] Huang C., Wang H., Ma S. (2021). J. Chromatogr. A.

[cit19] Hu R., Zhang X., Chi K.-N., Yang T., Yang Y.-H. (2020). ACS Appl. Mater. Interfaces.

[cit20] Ikigaki K., Okada K., Tokudome Y., Toyao T., Falcaro P., Doonan C. J., Takahashi M. (2019). Angew. Chem..

[cit21] Li J., Zhou Y., Sun Z. (2020). J. Chromatogr. A.

[cit22] Liu L., Qiao Z., Cui X., Pang C., Liang H., Xie P., Luo X., Huang Z., Zhang Y., Zhao Z. (2019). ACS Appl. Mater. Interfaces.

[cit23] Amirzehni M., Hassanzadeh J., Vahid B. (2020). Sens. Actuators, B.

[cit24] Mao X., Yan A., Wan Y., Luo D., Yang H. (2019). J. Agric. Food Chem..

[cit25] Tanhaei M., Mahjoub A. R., Safarifard V. (2018). Ultrason. Sonochem..

[cit26] Li Y., Shen Y., Zhang Y., Zeng T., Wan Q., Lai G., Yang N. (2021). Anal. Chim. Acta.

[cit27] Saleem H., Rafique U., Davies R. P. (2016). Microporous Mesoporous Mater..

[cit28] Aghili F., Ghoreyshi A. A., Rahimpour A., Van der Bruggen B. (2020). Ind. Eng. Chem. Res..

[cit29] Zhu J., Hou J., Yuan S., Zhao Y., Li Y., Zhang R., Tian M., Li J., Wang J., Van der Bruggen B. (2019). J. Mater. Chem. A.

[cit30] Hou Y., Jiang X., Gao Y., Li Y., Huang W., Chen H., Tang X., Tsunoda M., Li J., Zhang Y. (2021). Microchem. J..

[cit31] Fang X., Wu S., Wu Y., Yang W., Li Y., He J., Hong P., Nie M., Xie C., Wu Z. (2020). Appl. Surf. Sci..

[cit32] Liu W., Zhang J., Zhang C., Ren L. (2011). Chem. Eng. J..

[cit33] Pei Z.-G., Shan X.-Q., Zhang S.-Z., Kong J.-J., Wen B., Zhang J., Zheng L.-R., Xie Y.-N., Janssens K. (2011). J. Hazard. Mater..

[cit34] Chi H., Li C., Huang M. (2021). Chem. Eng. J..

[cit35] Sun J., Guo W., Ji J. (2020). LWT.

[cit36] Chen W., Li X., Pan Z., Bao Y., Ma S., Li L. (2015). Chem. Eng. J..

[cit37] Zhang Y., Xie Y., Zhang C., Wu M., Feng S. (2020). J. Sep. Sci..

[cit38] Liang D., Wang X., Liu J., Liu J., Tang S., Xu B., Jin R. (2022). J. Mol. Graphics Modell..

[cit39] Benito-Peña E., Martins S., Orellana G., Moreno-Bondi M. C. (2009). Anal. Bioanal. Chem..

[cit40] Lu W., Liu J., Li J., Wang X., Lv M., Cui R., Chen L. (2019). Analyst.

[cit41] Barahona F., Albero B., Tadeo J. L., Martín-Esteban A. (2019). J. Chromatogr. A.

