# Supplementary material for: Synthesis and evaluation of UiO-66@MIP towards norfloxacin in water
Source: RSC Adv. 2022 Jul 21;12(32):20785–91. doi: 10.1039/d2ra02726g (PMC9301542; doi:10.1039/d2ra02726g)
Supplement: RA-012-D2RA02726G-s001 [file RA-012-D2RA02726G-s001.pdf]

Electronic Supplementary Material (ESI) for RSC Advances.
This journal is © The Royal Society of Chemistry 2022

Supporting Information

Removal and detection of norfloxacin in water by UiO-66@MIP

Wu Zixuan[1], Zhang Sixue[1], Liu Wanqiong[2,3], Peng Zhihua[2], Dong Yanshan[2], Huang Zeyu[2], Zhong Mingmin[1], Liang Yong[1,*], Ye Youai[1]

[1] Department of Analycital Chemistry, Faculty of Chemistry, South China Normal University, Guangzhou, China

[2] Foshan Sanshui Foshui Water Supply Co., Ltd, Foshan, China

*Corresponding author: Dr. Liang Yong

E-mail addresses: liangy@scnu.edu.cn

1. Synthesis of MIP without UiO-66-NH_2

51 mg of NOR and 68 μL of MAA were dissolved into 50 mL of acetonitrile. The mixture was stirred for 2 h at room temperature. After the reaction system was heated to 60 °C, 400 μL EGDMA and 70 mg AIBN were added to the solution. The mixture reacted at 60 degrees for 24 hours. After the reaction, the precipitate was collected with a centrifuge at 9000 rpm, then washed with methanol /acetic acid (90:10, v/v) until the template was removed. Finally, the product was dried at 60 °C under vacuum.

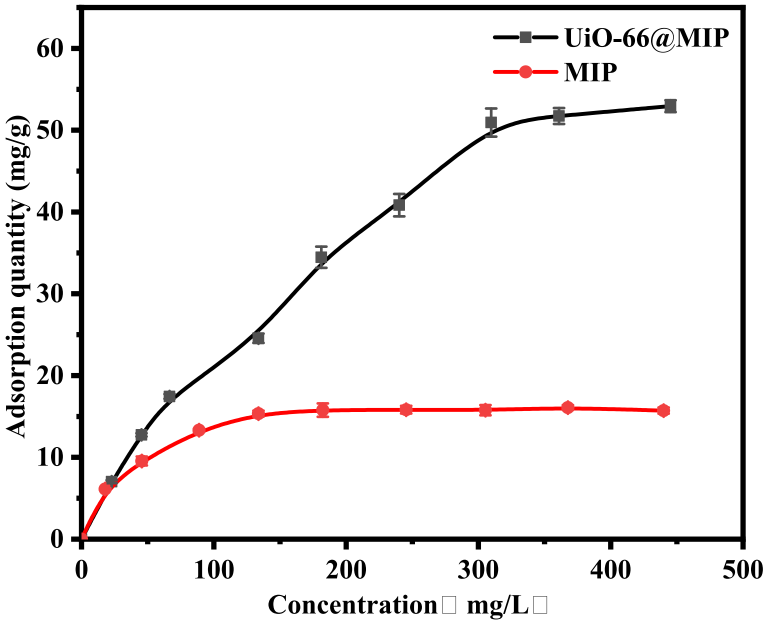

Figure S1. The static adsorption curves of UiO-66@MIP and MIP without UiO-66-NH_2

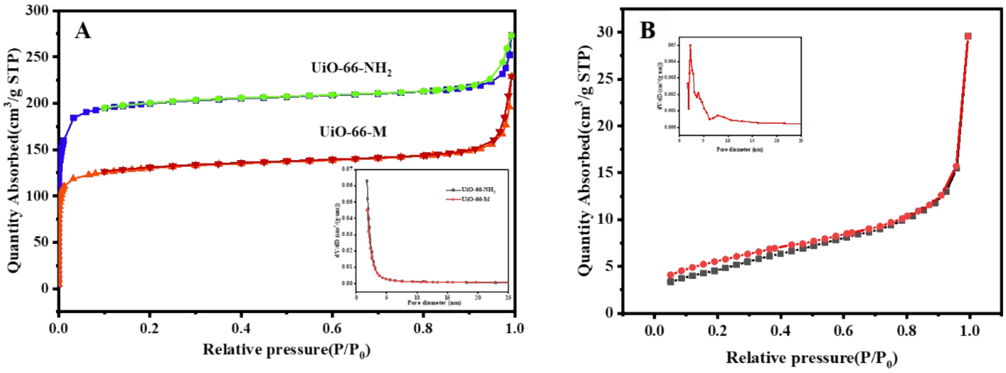

Figure S2. (A) N_2 sorption isotherms of UiO-66-NH_2 and UiO-66-M and their pore sizes distribution; (B) N_2 sorption isotherms of UiO-66@MIP and its pore sizes distribution

Table S1 Isotherm parameters obtained by the Langmuir and Freundlich isotherm models.

Materials	UiO-66@NIP	UiO-66@MIP
**Freundlich**		
1/n	0.56	0.42
Log K_F	0.48	0.58
R^2	0.98	0.98
**Langmuir**		
Q_m (mg/g)	26.9	64.3
K_L	0.037	0.010
R^2	0.91	0.95

Table S2 Results of UiO-66@MIP applied in real water sample

Water sample	Spiked concentration (mg/L)	Measured concentration (mg/L)	Recovery (%)	RSD (%, n=3)
1	0	0.45	/	1.9
2	1	1.43	98.1	2.1
3	3	3.40	98.3	5.5
4	5	5.29	96.7	4.4
